# Elevated TAB182 enhances the radioresistance of esophageal squamous cell carcinoma through G2‐M checkpoint modulation

**DOI:** 10.1002/cam4.3879

**Published:** 2021-03-30

**Authors:** Yuandong Cao, Aidi Gao, Xiaoqing Li, Han Min, Chao He, Xinchen Sun, Wei‐Qun Ding, Jundong Zhou

**Affiliations:** ^1^ Department of Radiation Oncology The First Affiliated Hospital of Nanjing Medical University Nanjing Jiangsu P.R. China; ^2^ Suzhou Cancer Center Core Laboratory The Affiliated Suzhou Hospital of Nanjing Medical University Suzhou Jiangsu P.R. China; ^3^ Department of Pathology University of Oklahoma Health Science Center Oklahoma City OK USA

**Keywords:** esophageal squamous cell carcinoma, FHL2, G2‐M cell cycle checkpoint, radioresistance, TAB182

## Abstract

**Background:**

Radiotherapy is one of the main strategies for the treatment of esophageal squamous cell carcinoma (ESCC). However, treatment failure often occurs due to the emergence of radioresistance. In this study, we report a key regulator of radiation sensitivity, termed TAB182 that may become an ideal biomarker and therapeutic target to overcome radioresistance.

**Materials and Methods:**

By applying qRT‐PCR and immunohistochemical staining, the expression of TAB182 was detected in patient tissues. We next assessed the influence of TAB182 downregulation to radiosensitivity using clonogenic survival assay and γ‐H2A.X foci analysis in TE‐1, TE‐10, and radioresistant TE‐1R cell lines after ionizing radiation. To unveil the mechanism underlying, TAB182 interacting proteins were identified by mass spectrometry following co‐immunoprecipitation. Furthermore, flow cytometry and western blot assay were applied to validate the identified proteins.

**Results:**

Our results demonstrated that the expression of TAB182 is higher in cancer tissues than normal tissues and elevated expression of TAB182 correlates with poor outcomes of postoperative radiotherapy. Downregulation of TAB182 sensitized cancer cells to ionizing radiation, particularly in radioresistant TE‐1R cells that spontaneously overexpress TAB182. Mechanically, TAB182 interacts with FHL2 to induce G2‐M arrest through wiring the CHK2/CDC25C/CDC2 signaling pathway. Finally, overexpression of shRNA‐resistant TAB182 restored the checkpoint and radioresistance.

**Conclusion:**

TAB182 potentiates the radioresistance of ESCC cells by modulating the G2‐M checkpoint through its interaction with FHL2. Thus, TAB182 may become an ideal biomarker and therapeutic target of ESCC radiotherapy.

## Graphical abstract

Our findings suggest a novel mechanism of radioresistance of ESCC in which TAB182 modulates G2‐M checkpoint to potentiate ESCC radioresistance. Therefore, TAB182 may serve as an ideal biomarker to predict prognosis of postoperative radiotherapy and may become a potent therapeutic target to sensitize ESCC to radiotherapy.

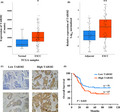

## 1 INTRODUCTION

Esophageal cancer is ranked the ninth most common cancer and the sixth leading cause of cancer death worldwide.^1^, ^2^ The 5‐year relative survival rate is only 19%, which is the third‐lowest in all cancers.^3^ The major histological type is esophageal squamous cell carcinoma (ESCC), which accounts for about 80% of all esophageal cancers.^4^ In China, ESCC is the fourth leading cause of estimated cancer deaths.^5^ The high mortality rate of ESCC calls for more effective treatment of the disease, one of the main treatment options for ESCC is radiotherapy. However, a major problem with radiotherapy is the treatment failure caused by radioresistance.^6^ Thus, there is an urgent need for identifying the novel therapeutic target to overcome radioresistance.

Great efforts have been devoted to investigating the mechanisms underlying radioresistance. Mounting evidence suggested that one of the main factors contributing to radioresistance is cell cycle arrest, especially at the G2‐M phase.^6^, ^7^, ^8^ This critical G2‐M checkpoint process provides cells enough time to repair damaged DNA. After ionizing radiation (IR), checkpoint kinase 2 (CHK2) is rapidly activated by ataxia‐telangiectasia mutated gene (ATM serine/threonine kinase, ATM) and inactivate the phosphatase cell division cycle 25C (CDC25C) by phosphorylation.^9^, ^10^ Then, four and a half LIM domains 2 (FHL2), together with tyrosine 3‐monooxygenase/tryptophan 5‐monooxygenase activation protein theta (YWHAQ or 14‐3‐3), forms a complex with phosphorylated CDC25C and sequesters the latter in the cytoplasm; the inactivated CDC25C fails to dephosphorylate Cell Division Cycle 2 (CDC2), leading to G2‐M phase arrest.^11^, ^12^, ^13^, ^14^

TAB182, also called Tankyrase‐binding protein 1 (TNKS1BP1), was identified interacting with tankyrase‐1 through the RXXPDG motif.^15^, ^16^ TAB182 was reported to locate at nucleus and cytoplasm.^15^ In the cytoplasm, TAB182 interacted with the multi‐subunit CCR4 (carbon catabolite repressor 4)‐NOT (Negative on TATA) complex (CCR4‐NOT complex, CNOT) to affect the outcome of target mRNA by modulating helicase recruitment to the complex.^17^ Besides, TAB182 also regulated cancer cell invasion through actin cytoskeleton rearrangement modulation.^18^ Moreover, investigators have recently examined the functions of TAB182 in DNA damage repair after ionizing radiation or adenovirus infection,^19^, ^20^ suggesting a possible role of TAB182 in the development of radioresistance. Here in the present study, by analyzing patient samples, TAB182 was found to be elevated in ESCC tissues and negatively correlated with poor prognosis of postoperative radiotherapy. Mechanically, TAB182 interacted with FHL2 to induce G2‐M arrest through wiring the CHK2/CDC25C/CDC2 signaling pathway. These novel findings indicate that TAB182 may serve as a potential biomarker and therapeutic target of radiotherapy.

## 2 MATERIALS AND METHODS

### 2.1 Tissue samples and cell lines

All patient samples were immediately snap‐frozen and stored at −80℃ for immunohistochemical study. Human ESCC cell lines of TE‐1 and TE‐10 were kindly provided by Stem Cell Bank, Chinese Academy of Sciences. TE‐1 and TE‐10 cells were grown in DMEM or RPMI‐1640 medium supplemented with 10% FBS. All cells were incubated in a humidified atmosphere with 5% CO_2_ at 37℃.

### 2.2 RNA extraction and quantitative RT‐PCR

Total RNA was isolated using TRIzol Reagent (Invitrogen Life Technologies). The concentrations of RNA were determined by Nano‐Drop2000 (Thermo Scientific). For reverse transcription, 1 μg of total RNA was reverse transcribed by Revert‐Aid First Strand cDNA Synthesis Kit (Thermo Scientific). Quantitative PCR analyses were conducted to measure mRNA expression level by Quanti‐Nova SYBR Green PCR Kit (QIAGEN), with β‐actin mRNA level served as an internal control. Quantitative PCR was performed using a StepOnePlus instrument (Applied Biosystems). The primers for TAB182 were: forward, 5ʹ‐ GCTTATGCCAGCCAAGATGC‐3ʹ; reverse, 5ʹ‐CGCTCTTCCCAAACTCCTGT‐3ʹ. The primers for β‐actin were: forward, 5ʹ‐CACCATTGGCAATGAGCGGTTCC‐3ʹ; reverse, 5ʹ‐GTAGTTTCGTGGATGCCACAGG‐3ʹ.

### 2.3 Immunohistochemical analysis

Expression of TAB182 in human ESCC and adjacent tissues were analyzed using immunohistochemistry. Briefly, Tumor tissue sections were deparaffined in xylene, followed by rehydration using ethanol gradient from 100%, 95% to 75%. Epitope retrieval was performed using citrate buffer pH6.0 and heat‐treated for 5 min. The tissue sections were then exposed in 0.03% hydrogen peroxide for 5 min to block endogenous peroxidase, after which sections were incubated with 2% BSA for 30 min. Then, sections were incubated with the primary antibody of TAB182 (ab155498, Abcam) overnight at 4℃. HRP‐conjugated anti‐rabbit secondary antibody (GAR0072, Multisciences) was then added for 1 h and the color was developed by 3‐3ʹ‐diaminobenzidine. Then the sections were counterstained with hematoxylin, washed, and dipped briefly in a water bath containing drops of ammonia, before dehydration and mounting in Diatex. The stained sections were analyzed and scored using a Leica microscope (Leica). Scoring was based on the intensity and average percentage of positive cells. The staining intensity was scored with “1” (negative or weakly positive), “2” (moderately positive), and “3” (strongly positive). The average percentage of positive cells was scored as 1 (<25%), 2 (25–50%), 3 (50–75%), and 4 (>75%). The score was calculated by multiplying the score of staining intensity and percentage of positive cells. The scores ≥6 were considered of higher expression, while scores <6 were considered of lower expression.

### 2.4 Stable cell line generation

TAB182 shRNA and negative control were cloned into the lentivirus vector LV3 (GenePharma). Cells were infected with lentivirus and selected by medium containing Puromycin (Sigma‐Aldrich) for 7 days. For rescue experiments, the RNA interference‐resistant coding region of TAB182 containing synonymous mutation was amplified and cloned into pcDNA3.1 (+) vector (GenePharma). Plasmid transfection was performed using lipofectamine 3000 (Invitrogen Life Technologies) following the manufacturer's instructions. Cells were selected by G‐418 (Sigma‐Aldrich) containing medium after 24 h of transfection for 10 days.

### 2.5 Clonogenic survival assay

Harvested cells were seeded in 6‐well plates at different densities: 2 × 10^2^, 1 × 10^3^, 2 × 10^3^, 6 × 10^3^, 1.2 × 10^4^, and 2 × 10^4^ cells per well. Cells were irradiated with doses of 0, 2, 4, 6, 8, 10 Gy, respectively, by a linear x‐ray accelerator (Varian) at the dose rate of 200 cGy/min. After 14 days of incubation, cells were stained with Wright‐Giemsa solution (Nanjing Jiancheng Bioengineering Technology). Colonies containing more than 50 cells were counted. The assay was repeated three times for each cell line. The planting efficiency (PE) equals to the number of colonies formed/number of cells seeded. A multitarget click model was applied as SF = 1 − (1 − e^−DK^)^N^. Then quasi‐threshold dose (*D*_q_) and mean lethal dose (*D*_0_) were calculated: *D*_q_ = LN(N)*D_0_; *D*_0_ = 1/K.

### 2.6 Immunofluorescent staining

Cells were irradiated with a dose of 4 Gy x‐ray. The distance to the radiation source was 100 mm. After irradiation, cells were fixed using 4% paraformaldehyde (Solarbio) for 10 min at room temperature. Then, fixed cells were permeated by 0.2% Triton X‐100 for 10 min, followed by blocking using 5% BSA for 1 h. Cells were incubated with primary antibody against γ‐H2A.X (ab81299, Abcam) overnight at 4℃. After wash, the cells were incubated with DyLight 649 goat anti‐rabbit IgG (H+L) secondary antibody (GAR6492, Multisciences) for 1 h at room temperature and then stained with DAPI containing antifading mounting medium (Solarbio).

### 2.7 Radio‐resistant cell line generation

Cells were irradiated with 4 Gy x‐ray. After 24 h, cells were washed with PBS and incubated in the fresh cultural medium for 6 days. Then, cells undergo another round of irradiation and incubation to a total dosage of 60 Gy, after which a single‐cell cloning process was carried out. Radio‐resistance of every clone was tested using clonogenic survival assay mentioned above.

### 2.8 Immunoprecipitation assay

The assay was performed using the Immunoprecipitation Kit (Abcam) following the manufacturer's instructions. In brief, cells were lysed with non‐denaturing Lysis Buffer with protease inhibitors. Antibodies (Anti‐TAB182, sc‐514517, Santa Cruz; anti‐FHL2, 21619‐1‐AP, Proteintech) were added and incubated on a rotary mixer for 1 h at 4℃. Rabbit or mouse IgG (Beyotime Biotechnology) was used as a negative control. Afterward, washed protein A/G Sepharose^®^ was added and incubated overnight on a rotary mixer at 4℃. After washing three times, the beads with antigen‐antibody complex were eluted using 2× SDS‐PAGE loading buffer and boil for five min. The eluent was collected and stored at −80℃ for further analysis.

### 2.9 In‐gel digestion and LC–MS/MS analysis

TAB182 interacting proteins were analyzed after immunoprecipitation by PTM‐Biolabs Co., Ltd. For in‐gel tryptic digestion, gel pieces were destained in 50 mM NH_4_HCO_3_ in 50% acetonitrile (v/v) until clear. After dehydration with 100% acetonitrile and rehydration with 10 mM dithiothreitol and 55 mM iodoacetamide (Sigma‐Aldrich), gel pieces were digested with 10 ng/μl of trypsin resuspended in 50 mM NH_4_HCO_3_ overnight at 37℃. Peptides were extracted with 50% acetonitrile/5% formic acid and 100% acetonitrile, followed by drying to completion and resuspending in 2% acetonitrile/0.1% formic acid. For LC–MS/MS analysis, peptides were loaded onto a reversed‐phase analytical column and analyzed by tandem mass spectrometry (MS/MS) in Q Exactive^TM^ Plus (Thermo Scientific)‐coupled online to an EASY‐nLC 1000 UPLC system (Thermo Scientific). The resulting MS/MS data were processed using Proteome Discoverer 1.3 software (Thermo Scientific).

### 2.10 Cell cycle analysis

Mitotic cells were analyzed using anti‐phospho‐histone H3 (Ser 10) antibody (YP0129, ImmunoWay) and PI double staining. Briefly, cells were fixed by ice‐cold 75% ethanol overnight. Afterward, cells were incubated with anti‐phospho‐histone H3 (Ser 10) antibody for 1 h. After washing with PBS, cells were immunofluorescently labelled with DyLight 649 goat anti‐rabbit IgG (H+L) secondary antibody (Multisciences) containing PI and RNase A at 37℃ in the dark. Flow cytometry assay was performed using FACSCalibur (BD Biosciences). Results were analyzed by FlowJo^TM^ V10.

### 2.11 Western blot assay

Harvested cells were lysed using M‐PER^TM^ Mammalian Protein Extraction Reagent (Thermo Scientific) with protease and phosphatase inhibitors (Beyotime). Protein concentration was determined by BCA protein quantification kit (Beyotime). Equal amounts of the proteins were loaded into SurePAGEᵀᴹ precast polyacrylamide gels with a gradient between 4% and 20% (GenScript) and transferred to PVDF membranes (Millipore) by eBlot^®^ L1 wet protein transfer system (GenScript). After blocking, the membranes were incubated with primary antibodies overnight at 4℃. Then, the membranes were incubated with HRP‐conjugated anti‐mouse or anti‐rabbit secondary antibody (GAM0072 or GAR0072, Multisciences). The protein bands were visualized by High‐sig ECL Western Blotting Substrate (Tanon). Images were collected using the Tanon‐5200 Chemiluminescent Imaging System (Tanon). The expression of β‐actin was detected as a loading control.

All primary antibodies were commercial products. Anti‐TAB182, anti‐CDC2 (sc‐137035), anti‐phospho‐CDC2 (sc‐136014) were purchased from Santa Cruz. Anti‐FHL2 was purchased from Proteintech. Anti‐CHK2 (ab207446), anti‐phospho‐CHK2 (ab32148) were purchased from Abcam; anti‐CDC25C (YM0142), anti‐phospho‐CDC25C (YP0058) were purchased from ImmunoWay. Anti‐β‐actin (ab008) was purchased from Multisciences.

### 2.12 Statistical analysis

The quantitative results are presented as the mean values ± SD. Statistical analyses were performed using SPSS 19.0 software (IBM). *p*‐value of <0.05 was considered statistical significance. Differences between groups were estimated using the Student's *t*‐test. The overall survival rate was calculated according to the Kaplan–Meier method and analyzed by the log‐rank test.

## 3 RESULTS

### 3.1 Elevated expression of TAB182 correlates with poor prognosis of postoperative radiotherapy

**FIGURE 1:**
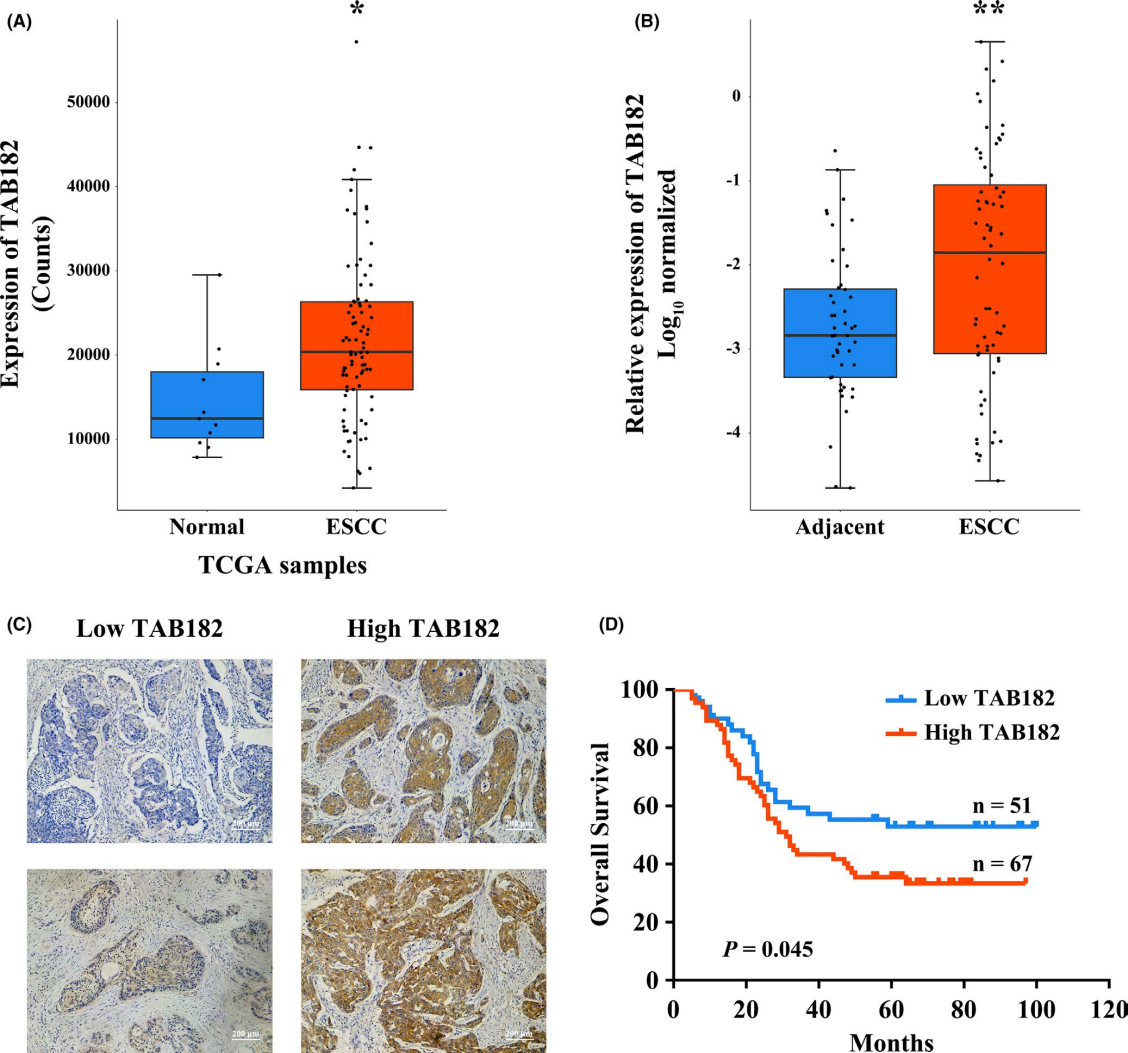
Expression of TAB182 in clinical samples. (A) The mRNA level of TAB182 in patient samples from the TCGA database and (B) from The Affiliated Suzhou Hospital of Nanjing Medical University. *p < 0.05, **p < 0.01. (C) Immunohistochemical staining of TAB182 in paraffin‐embedded ESCC tissues. (D) Kaplan–Meier survival analysis based on different IHC scores of TAB182

To investigate the expression of TAB182 in ESCC tissue, samples from the TCGA database were analyzed. As seen in Figure 1A, the median count of TAB182 in 88 ESCC samples was 20357, higher than that of 12445 in 11 normal samples, *p* = 0.01. Next, we validated the expression of TAB182 in tissue samples of 68 ESCC patients, and 48 of them were paired with adjacent samples. Similar results were observed showing that relative expression of TAB182 is higher in ESCC samples, *p* < 0.01 (Figure 1B, Figure S1).

Since TAB182 was proven to play a role in response to ionizing radiation and participated in DNA repair, we further investigated the correlation of its expression with prognosis in ESCC patients who received postoperative radiotherapy. Total of 118 cases was collected. Immunohistochemical staining was carried out to identify the expression of TAB182 (Figure S2). All cases were divided into higher expression and lower expression groups based on the IHC score of TAB182. As seen in Figure 1C, TAB182 located mostly in the cytoplasm. Next, Kaplan‐Meier survival analysis was employed to evaluate the survival between two groups. Results in Figure 1D suggested that patients with higher TAB182 expression had a lower survival rate. In contrast, patients with lower TAB182 expression had a better prognosis.

Taken together, these results indicate that TAB182 is elevated in ESCC tissues and may negatively affect postoperative radiotherapy.

### 3.2 Downregulation of TAB182 increases the radiosensitivity of ESCC cell lines

**FIGURE 2:**
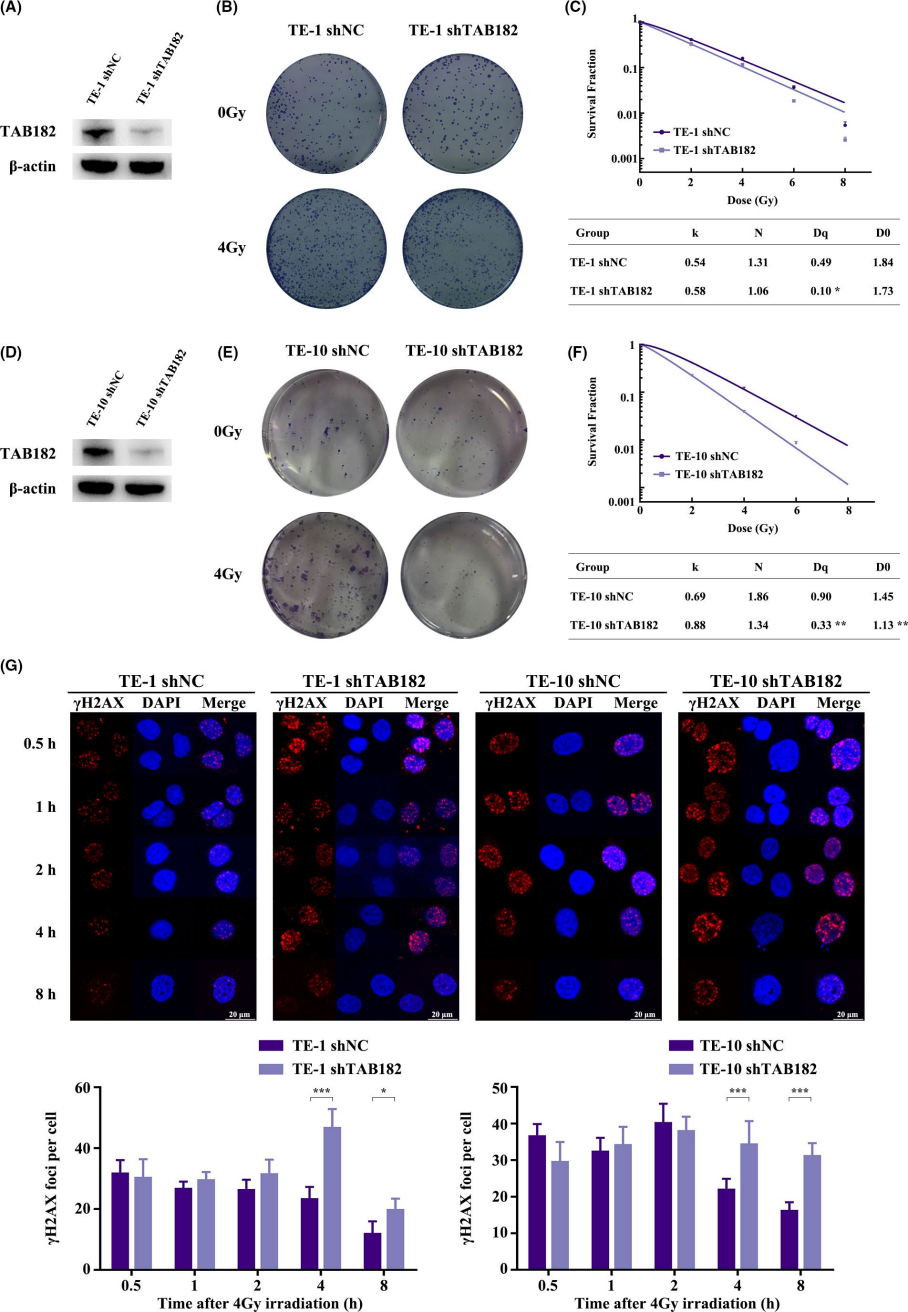
Radiosensitivity of ESCC cells after TAB182 downregulation. Western blot confirmed TAB182 downregulation in TE‐1 (A) and TE‐10 (D) cells. Clonogenic survival assay of TE‐1 (B) and TE‐10 cells (E) after ionizing radiation (*n* = 3). (C) and (F) Multitarget click model analysis after IR. Surviving fraction (SF) =1 − (1 − e^−DK^) ^N^. The quasi‐threshold dose (*D*_q_) and the mean lethal dose (*D*_0_) were calculated: *D*_q_ = LN(N) *D_0_; *D*_0_ = 1/*K*. (G) Immunofluorescent staining of γ‐H2A.X foci in control group and TAB182 downregulated group in TE‐1 and TE‐10 cells at different time points after 4 Gy IR. *p < 0.05, ***p < 0.001

To understand its influence on radiosensitivity, TAB182 expression was downregulated in ESCC cell lines TE‐1 and TE‐10 (Figure 2A and D). We then performed the clonogenic survival assay to evaluate the effect of TAB182 down‐regulation on radiosensitivity. As seen in Figure 2B and E and Figure S3A and B, in both cell lines, the downregulation of TAB182 led to less colony formation when irradiated. The assay results were analyzed using the multitarget click model. To quantitively describe the effect, quasi‐threshold dose (D_q_) and mean lethal dose (*D*_0_) were calculated. In this model, *D*_q_ represents the capability to accumulate sublethal damage and a lower *D*_q_ indicates an enhanced radiosensitivity. Similarly, a lower D_0_ indicates an elevated radiosensitivity. In Figure 2C, TAB182 downregulated TE‐1 cells showed lower *D*_q_ (0.10) than the control group (0.49). Likewise, *D*_q_ and *D*_0_ dropped from 0.90 to 1.45 in the control group to 0.33 and 1.13 in TAB182 downregulated TE‐10 cells (Figure 2F).

We next applied immunofluorescence microscopy to analyze the DNA double‐strand breakage (DSB). As seen in Figure 2G, γ‐H2A.X foci per cell in the control group was significantly lower at 4‐ and 8‐h post‐irradiation compared with TAB182 downregulated group in both cell lines.

Taken together, these observations show that the downregulation of TAB182 increases the radiosensitivity of ESCC cells.

### 3.3 TAB182 expression is upregulated in radioresistant ESCC cells

**FIGURE 3:**
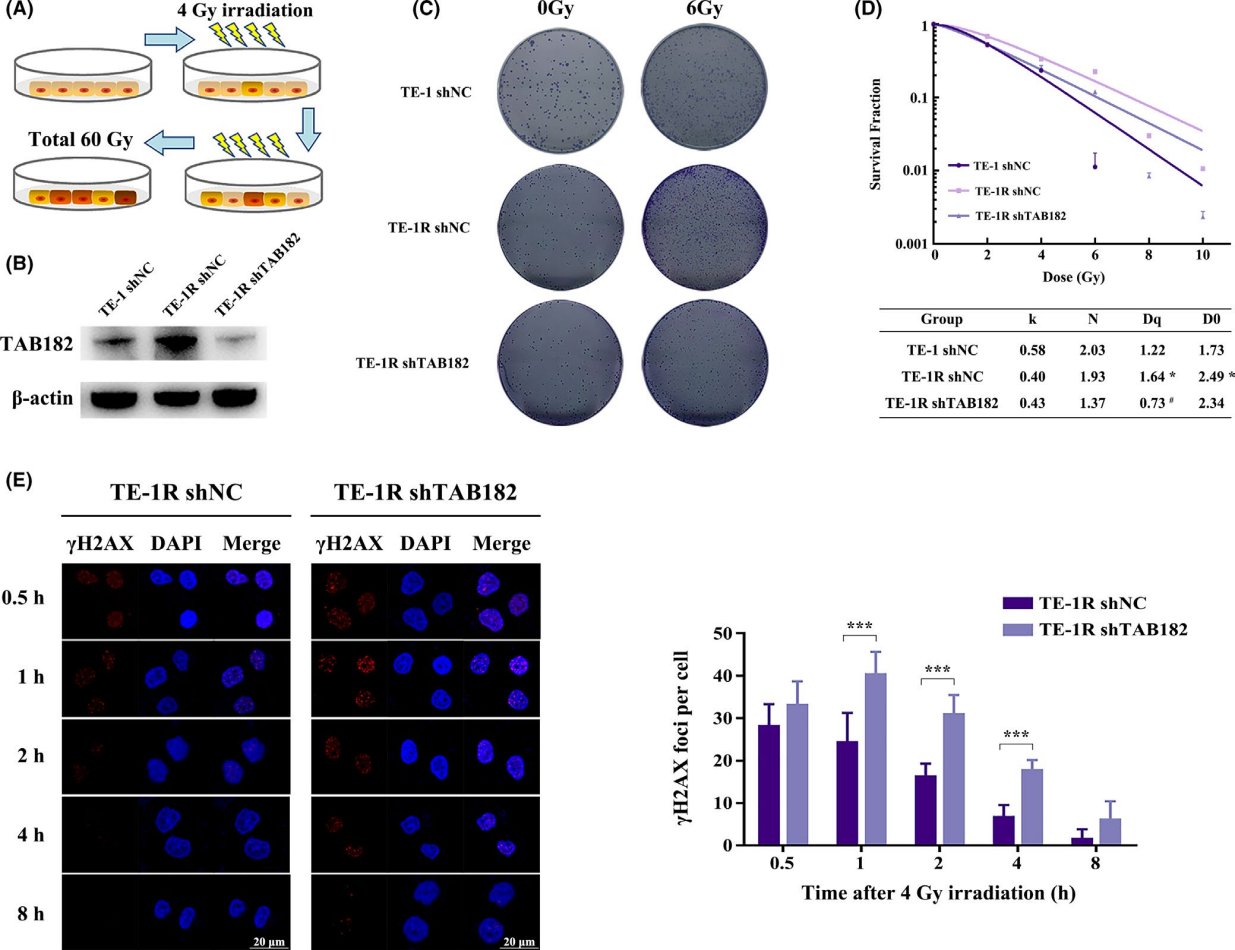
TAB182 affects radioresistance in radioresistant TE‐1R cells. (A) Scheme of radioresistant TE‐1R cells generation. (B) Western blot confirmed TAB182 upregulation in TE‐1R cells compared with parent TE‐1 cells and TAB182 expression was successfully downregulated by shRNA. (C) Clonogenic survival assay of TE‐1, TE‐1R, and TAB182 knockdown TE‐1R cells after ionizing radiation (*n* = 3). (D) Parameters of multitarget click model analysis after IR. (E) Immunofluorescent staining of γ‐H2A.X foci in the control group and TAB182 knockdown group at different time points after 4 Gy IR. *p < 0.05, ***p < 0.001 versus parent TE‐1 cells. ^#^ *p* < 0.05 versus control TE‐1R cells

To generate the radioresistant ESCC cells, TE‐1 cells were irradiated with 4 Gy x‐ray every 7 days until a total dosage of 60 Gy (Figure 3A). After single‐cell cloning assay, a radioresistant strain was selected, termed TE‐1R. Interestingly, the expression of TAB182 was significantly elevated in this strain (Figure 3B).

We next studied the effect of TAB182 on radioresistance in TE‐1R. Results in Figure 3C and Figure S3C confirmed that TE‐1R is more radioresistant than the parent cell line TE‐1 since more colonies formed after 6 Gy IR. In the meantime, downregulation of TAB182 lowered the numbers of colony formed, suggesting that the expression of TAB182 may be a key factor of radioresistance. This is supported by the multitarget click model (Figure 3D). TE‐1R cells exhibited higher survival fraction than parent cells, while TAB182 downregulation reduced the survival fraction. Both *D*_q_ and *D*_0_ were increased in TE‐1R cells, suggesting increased radioresistance. In contrast, *D*_q_ and *D*_0_ were decreased in TAB182 knockdown TE‐1R cells. Similar to its parent cells, TE‐1R cells showed more γ‐H2A.X foci per cell when TAB182 was downregulated (Figure 3E). It is worth noting that the foci of γ‐H2A.X per cell were decreased more rapidly in TE‐1R cells than in parent cells.

It is clear that TAB182 plays a critical role in radioresistant TE‐1R cells.

### 3.4 TAB182 interacts with FHL2 to modulate radiosensitivity through IR‐induced G2‐M arrest

**FIGURE 4:**
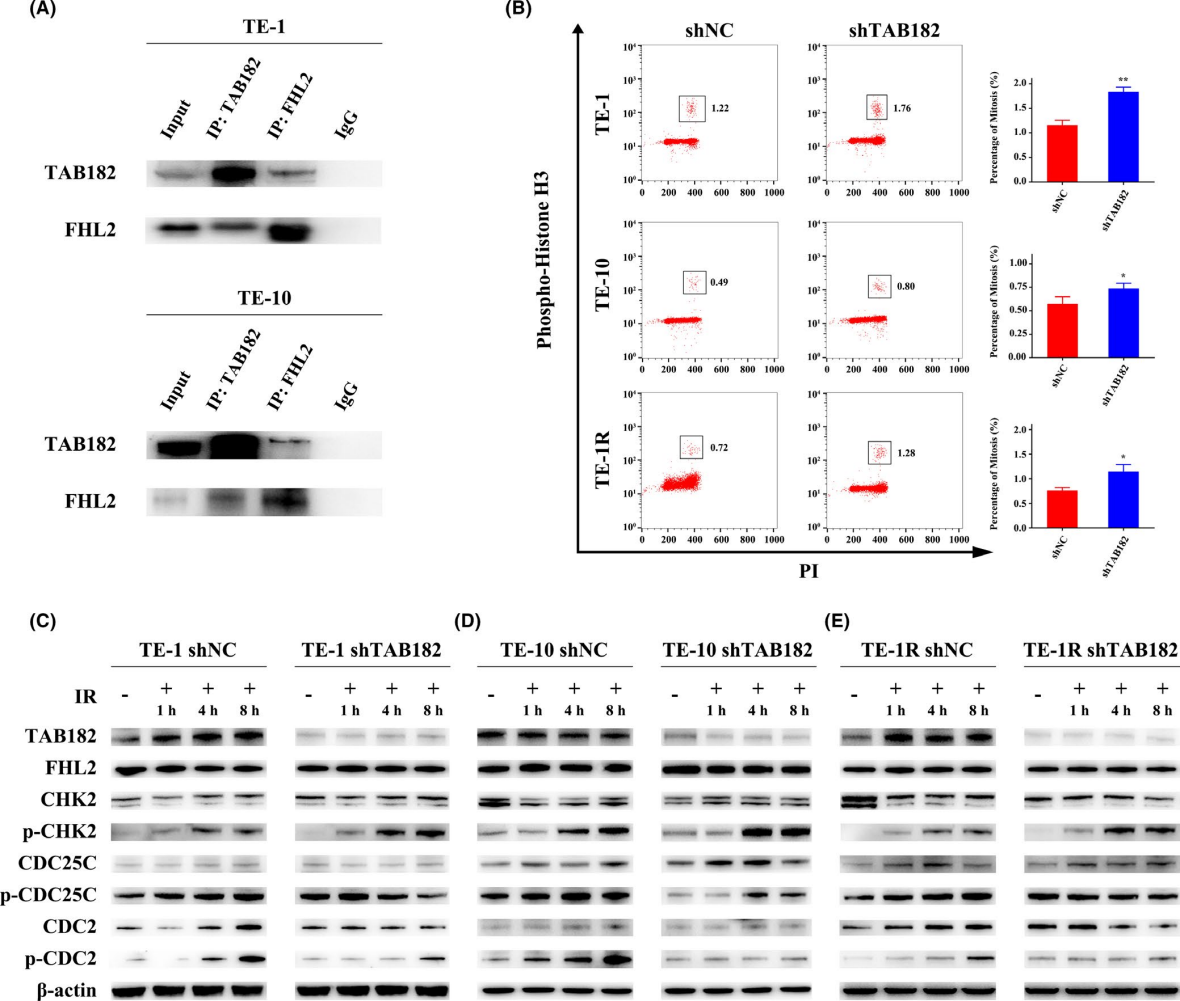
TAB182 regulates the G2‐M checkpoint through interaction with FHL2. (A) Co‐IP confirmed the interaction between TAB182 and FHL2 in both TE‐1 and TE‐10 cells. Mouse or rabbit IgG was used as a negative control. (B) The proportion of mitotic cells after 8 h of 4 Gy IR were analyzed by flow cytometry using the mitotic marker phospho‐histone H3 (Ser 10) and PI double staining. The mitotic cells were marked in the box. Western blot was employed to study the activation of the G2‐M checkpoint in TE‐1 (C), TE‐10 (D), and TE‐1R (E) cells. Cells were irradiated by 4 Gy IR and were harvested at different time points. The expression of β‐actin was determined as a loading control. *p < 0.05, **p < 0.01

To unveil the mechanisms of how TAB182 modulate radiosensitivity, a co‐immunoprecipitation‐coupled mass spectrometry was carried out to identify its interacting protein partners. Table 1 lists the identified interacting proteins in both TE‐1 and TE‐10 cells. In line with other studies, most components of the CNOT complex and subunits of capping actin protein of muscle Z‐line (CAPZA1, CAPZA2, and CAPZB) were on the list.^17^, ^18^, ^20^ Of note, FHL2, the critical regulator of G2‐M checkpoint and radiosensitivity, was found to interact with TAB182. Further Co‐IP analysis confirmed their interaction in both cell lines (Figure 4A, Figure S6). As expect, FHL2 downregulation sensitized TE‐1R cells to IR (Figure S7).

**TABLE 1:** Protein identified by mass spectrometry

Protein	TE‐1			TE‐10		
	No. of peptides	Coverage (%)	Protein score	No. of peptides	Coverage (%)	Protein score
TAB182	68	56.22	4224.1	47	34.76	2440.7
CNOT1	62	31.4	2836.2	43	20.41	1519.4
CNOT10	14	23.25	964.4	10	17.34	596.2
CNOT2	10	24.81	489.0	7	21.11	297.2
CNOT11	10	25.29	455.8	5	16.27	201.9
CNOT7	8	46.67	450.3	5	22.46	259.8
CNOT3	8	12.35	435.2	7	10.49	233.9
CNOT9	6	26.09	305.3	8	29.1	311.1
CAPZA1	11	55.59	868.4	8	41.61	393.7
CAPZA2	7	36.01	311.8	3	12.24	134.2
CAPZB	9	37.55	443.0	7	27.44	275.4
FHL2	12	52.33	623.9	2	10.39	82.0

To confirm whether the TAB182–FHL2 complex modulates the G2‐M checkpoint, flow cytometry assay was carried out with mitosis marker phospho‐histone H3 and PI double staining. Cells were harvested 8 h after irradiation and were fixed by ice‐cold 75% ethanol overnight. The cell cycle distribution is shown in Figure S4. After irradiation, cell cycle was arrested at the G2/M phase. As seen in Figure 4B, histone H3 phosphorylated mitosis cells were marked in the box. In all three cell lines, downregulation of TAB182 significantly increased the proportion of mitosis cells after IR, suggesting a possible deficiency of G2‐M checkpoint caused by TAB182 downregulation. However, the proportion of mitosis cells showed no significant change in the non‐irradiated group (Figure S5).

Next, we focused on the FHL2 regulated CHK2/CDC25C/CDC2 signaling pathway (Figure 4C–E). First of all, the expression of TAB182 was elevated in TE‐1 and TE‐1R control cells after irradiation, while it was kept unchanged in TE‐10 control cells. As expected, TAB182 expression was significantly lower and failed to increase in TE‐1 and TE‐1R cells with TAB182 knockdown. Second, no expression change in FHL2, CHK2, CDC25C, and CDC2 was observed. Third, when irradiated, phosphorylation of CHK2 significantly increased in all cells. Of note, it was even higher in TAB182 downregulated group. Last, phosphorylation of CDC25C and CDC2 was increased after irradiation in control cells. In contrast, in TAB182 downregulated cells, the phosphorylation either failed to increase or delayed.

Taken together, these data indicate that TAB182 downregulation hinders irradiation‐induced G2‐M arrest.

### 3.5 Overexpression of shRNA‐resistant TAB182 restores radioresistance and cell cycle checkpoint

**FIGURE 5:**
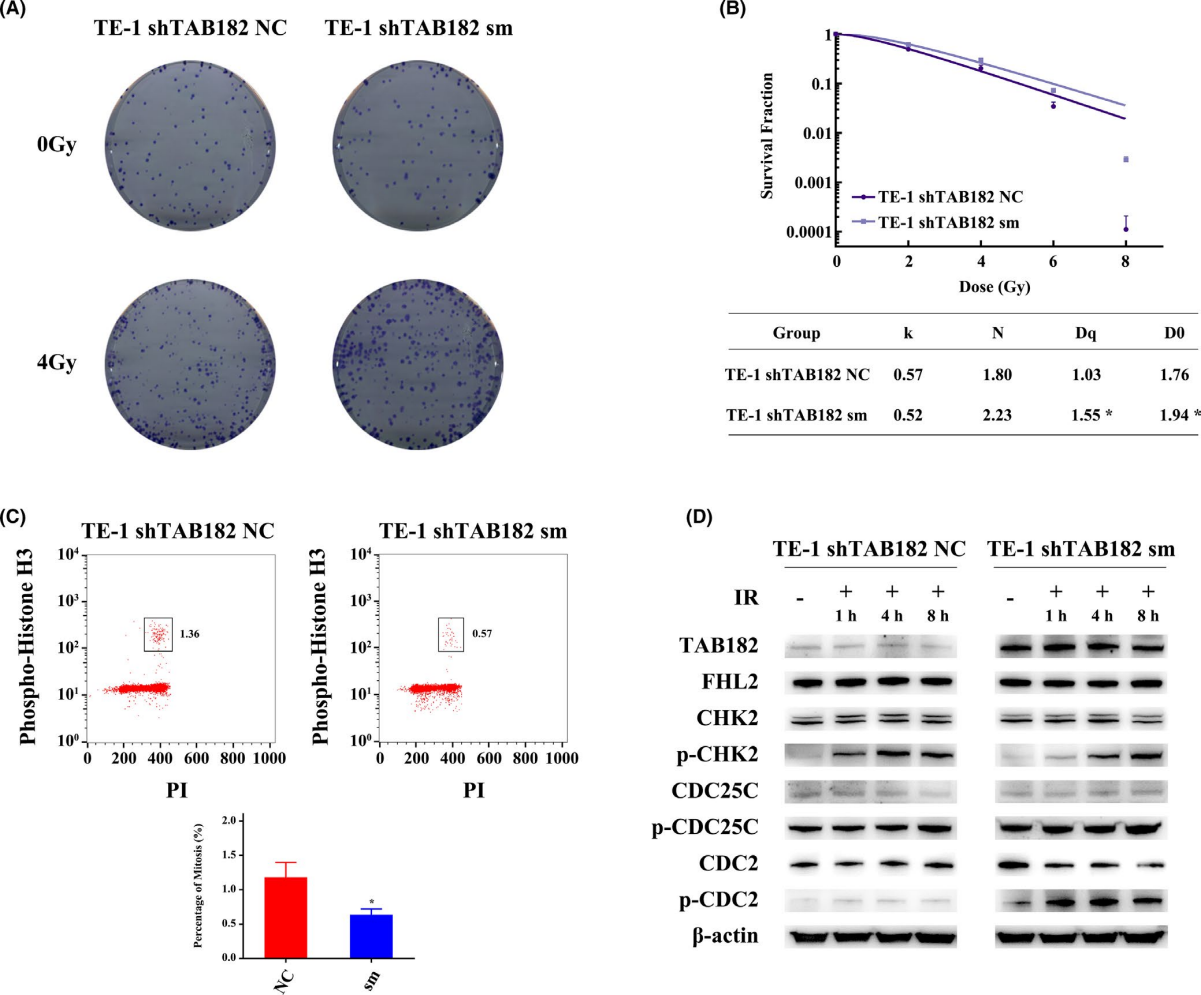
Overexpression of shRNA‐resistant TAB182 (synonymous mutation, sm) restores the G2‐M checkpoint in TAB182 downregulated TE‐1 cells. (A) Clonogenic survival assay after ionizing radiation (*n* = 3). (B) Parameters of multitarget click model analysis after IR. (C) The proportion of mitotic cells after 8 h of 4 Gy IR. (D) Western blot confirmed the restored G2‐M checkpoint after TAB182 overexpression. *p < 0.05

To further corroborate the role of TAB182 in radioresistance, rescue experiments were applied by upregulating TAB182 in TAB182 knockdown TE‐1 cells. The RNA interference‐resistant TAB182 coding region with the synonymous mutation was transfected into TE‐1 shTAB182 cells. Clonogenic survival assay confirmed that TAB182 overexpression restores the radioresistance, as indicated by more colony formation in TE‐1 shTAB182 sm than in the control group (Figure 5A, Figure S3D). Furthermore, the multitarget click model also supports the conclusion. As seen in Figure 5B, the survival fraction was higher in TE‐1 shTAB182 sm. The quasi‐threshold dose (*D*_q_) was higher in TAB182 upregulated cells (1.55) than that of the control group (1.03). The mean lethal dose (*D*_0_) also was increased. It reached 1.94 in the upregulated group from 1.76 in the control group.

Next, we analyzed the proportion of mitosis cells by flow cytometry. The results indicated that after irradiation, 1.36% of mitosis cells are detected in the control group, while the number reduces to 0.57% in TAB182 upregulated cells; nevertheless, no significant change was observed in the non‐irradiated group (Figure 5C, Figure S4, and Figure S5). In Figure 5D, western blot confirmed TAB182 upregulation. Similar to previous results, phospho‐CHK2 was increased after irradiation in both cells. Furthermore, both elevated phospho‐CDC25C and phospho‐CDC2 were observed in the TE‐1 shTAB182 sm group.

Taken together, we observed that the upregulation of TAB182 increases the radioresistance and restores the G2‐M checkpoint in our ESCC model systems.

## 4 DISCUSSION

Although distinct approaches have been developed to treat ESCC, there are obvious rooms left for improvement, such as more effective radiotherapy.^4^ Indeed, considering the high mortality rate of ESCC worldwide, there is a desperate need for identifying new biomarkers indicative of radiosensitivity or therapeutic targets that may sensitize radiotherapy. In this study, TAB182 expression was found to be elevated in ESCC tissues and correlated with poor prognosis of postoperative radiotherapy. Moreover, overexpression of TAB182 resulted in enhanced radioresistance by modulating the G2‐M checkpoint through interaction with FHL2.

Prior study has reported the overexpression of TAB182 in lung adenocarcinoma.^21^ Here in this study, by analyzing data from the TCGA database and patient samples from The Affiliated Suzhou Hospital of Nanjing Medical University, we also observed its overexpression in ESCC tissues. In addition, we found that patients with higher TAB182 expression exhibit poor outcomes when receiving postoperative radiotherapy. These observations indicate that TAB182 may serve as a predictive biomarker for postoperative radiotherapy. Further cellular analyses indicated that expression of TAB182 is elevated in the radioresistant TE‐1R cells, and TAB182 downregulation sensitizes wild‐type ESCC cells and radioresistant cells to ionizing radiation. Moreover, forced expression of TAB182 in TAB182 knockdown TE‐1 cells rescued the radioresistance. These results were confirmed by the multitarget click model in which TAB182 expression was positively correlated with quasi‐threshold dose (*D*_q_) and mean lethal dose (*D*_0_). Based on these results, we postulate that cancer cells may develop radioresistance when the expression of TAB182 is elevated.

Evidence have indicated that TAB182 may potentiate radioresistance through two distinct pathways after IR. On the one hand, TAB182 mediates the autophosphorylation of DNA‐PKcs, thus enhancing DNA damage repair activity,^19^ consistent with our observation showing that more γ‐H2A.X foci are detected after IR when TAB182 was downregulated in TE‐1 and TE‐10 cells. In radioresistant TE‐1R cells, similar results observed that there are more γ‐H2A.X foci in TE‐1R shTAB182 cells. Interestingly, fewer foci are seen in both control and TAB182 downregulated TE‐1R cells than that in TE‐1 cells. This may result from the elevated expression of genes involved in DNA repair such as Rad18 (data not shown). Downregulation of TAB182 may partially affect DNA repair capability.

On the other hand, we found that TAB182 wires the IR‐induced FHL2/CHK2/CDC25C/CDC2 signaling pathway through its interaction with FHL2, a key regulator of radioresistance.^11^, ^22^ It is known that under IR, FHL2 enhances the phosphorylation of the phosphatase CDC25C by forming a complex with CHK2 but not CHK1, making CDC25C inactivated and sequestered in the cytoplasm; and inactivated CDC25C fails to dephosphorylate CDC2, leading to the inactivation of CDC2 and subsequent G2‐M cell cycle arrest.^11^, ^12^ However, in our study, CHK2 activation was not affected by TAB182 downregulation. In addition, the phosphorylation of CHK2 was higher in the TAB182 knockdown group, consistent with increased γ‐H2A.X foci. Subsequent phosphorylation of CDC25C was failed due to TAB182 downregulation, indicating that TAB182 acts the downstream of CHK2 and functions with FHL2 to inactivate CDC25C. Thus, downregulation of TAB182 breaks the G2‐M checkpoint and results in an elevated proportion of M phase in ESCC cells after IR. This failed cell cycle arrest triggers mitotic catastrophe and apoptosis^11^ leading to enhanced radiosensitivity. However, the precise mechanism of the process remains obscure. Future works focusing on how TAB182 interacts with FHL2 and how the complex functions to facilitate cell cycle arrest may gain more insights into the mechanism of TAB182 induced radioresistance.

In conclusion, this study demonstrates that TAB182 potentiates radioresistance of ESCC cells by modulating G2‐M cell cycle arrest through its interaction with FHL2. This novel mechanism of radioresistance implies that TAB182 may become an ideal prognostic biomarker and therapeutic target of ESCC radiotherapy.

## CONFLICT OF INTEREST

The authors have no conflicts of interest to disclose.

## ETHICS STATEMENT

This study was approved by the Institutional Ethics Committee of Nanjing Medical University. Samples of human ESCC and adjacent tissues were obtained from The Affiliated Suzhou Hospital of Nanjing Medical University (Jiangsu, China) with informed consent.

## Supporting information

Fig S1

Fig S2

Fig S3

Fig S4

Fig S5

Fig S6

Fig S7

Supplementary Material

## Data Availability

Data available on request from the authors.
