# Supplementary figures and images for: Elevated TAB182 enhances the radioresistance of esophageal squamous cell carcinoma through G2‐M checkpoint modulation
Source: Cancer Med. 2021 Mar 30;10(9):3101–12. doi: 10.1002/cam4.3879 (PMC8085956; doi:10.1002/cam4.3879)

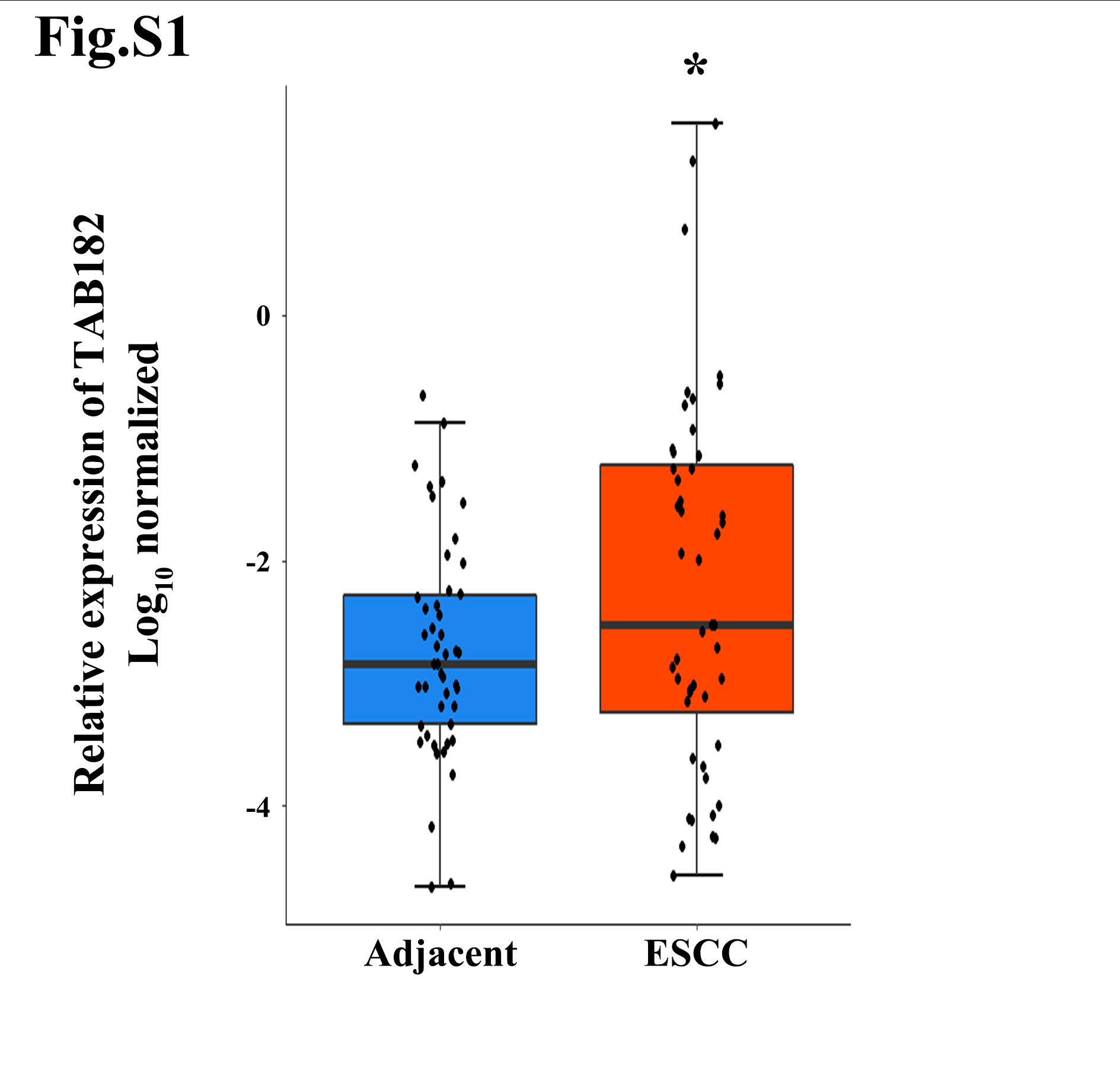

Supplement: Supplementary file 1 — Fig S1 [file CAM4-10-3101-s002.tif]

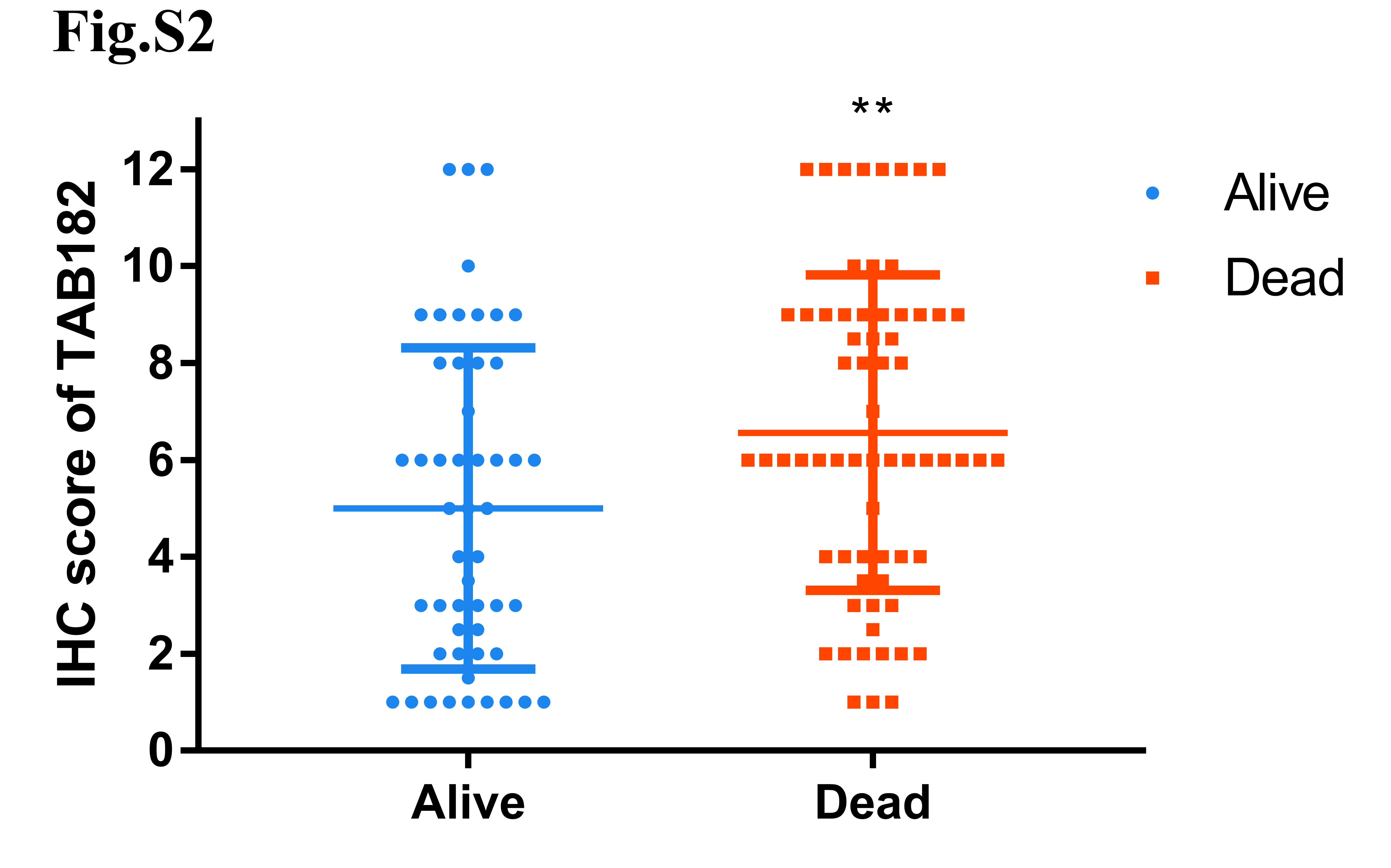

Supplement: Supplementary file 2 — Fig S2 [file CAM4-10-3101-s001.tif]

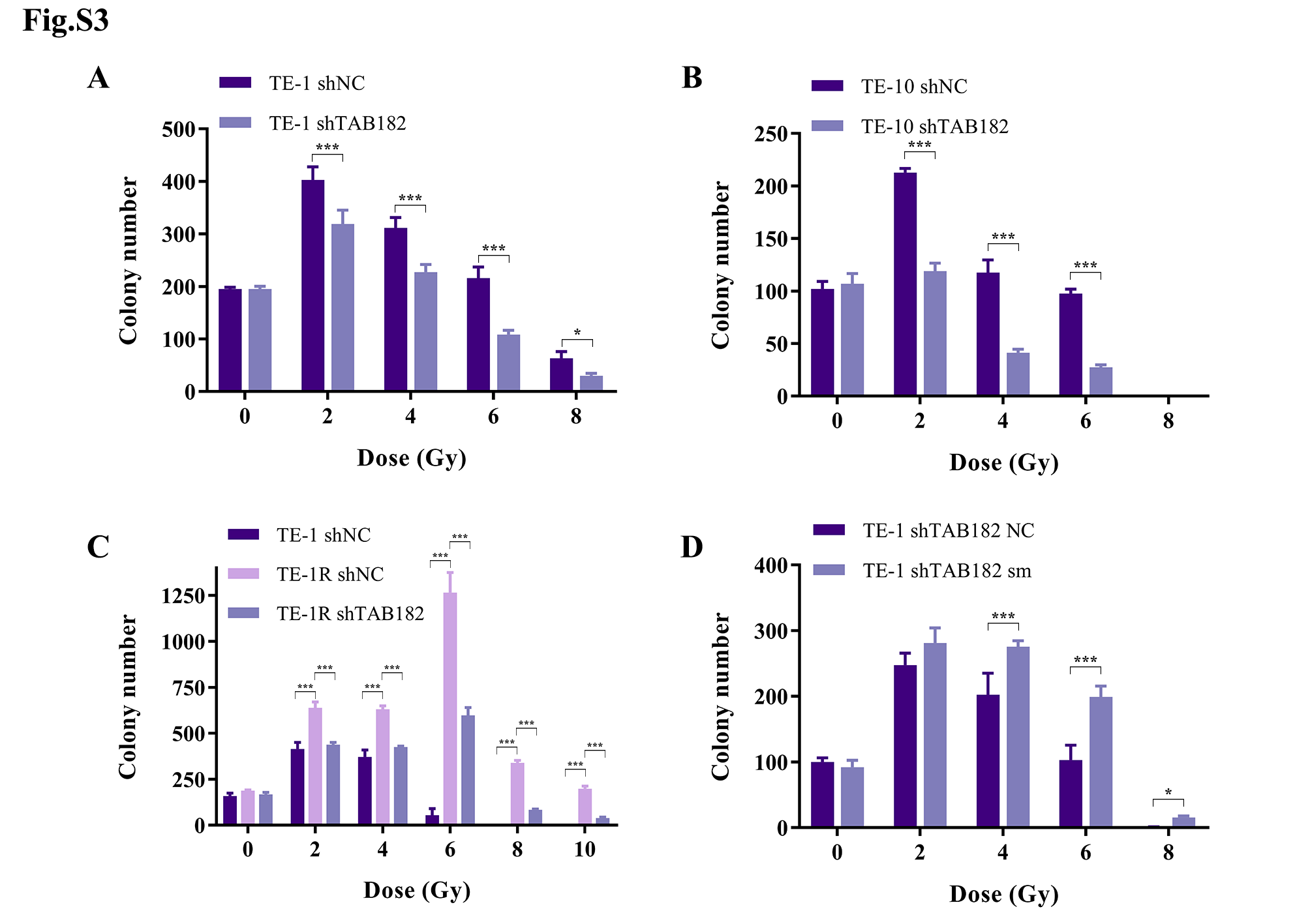

Supplement: Supplementary file 3 — Fig S3 [file CAM4-10-3101-s008.tif]

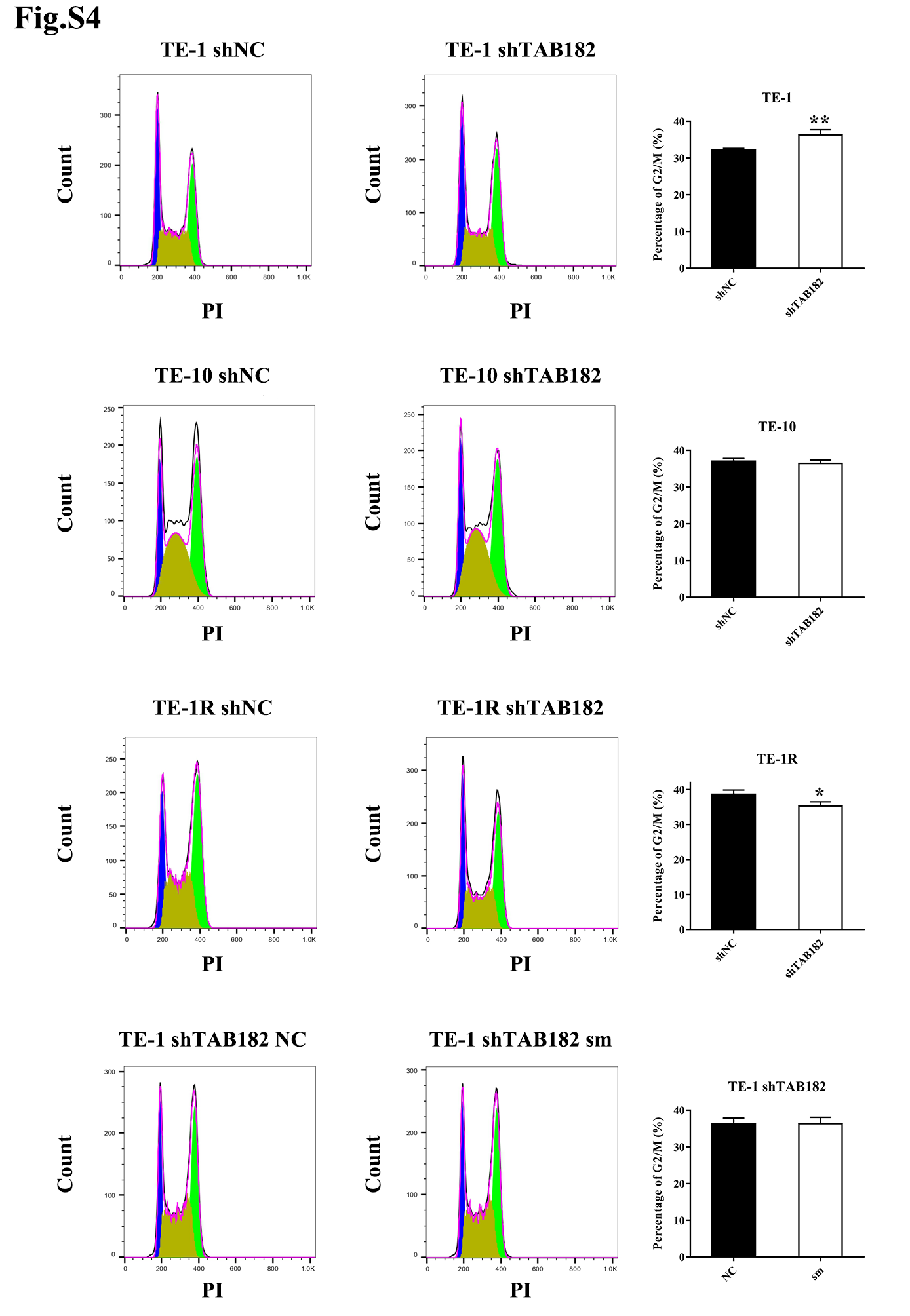

Supplement: Supplementary file 4 — Fig S4 [file CAM4-10-3101-s007.tif]

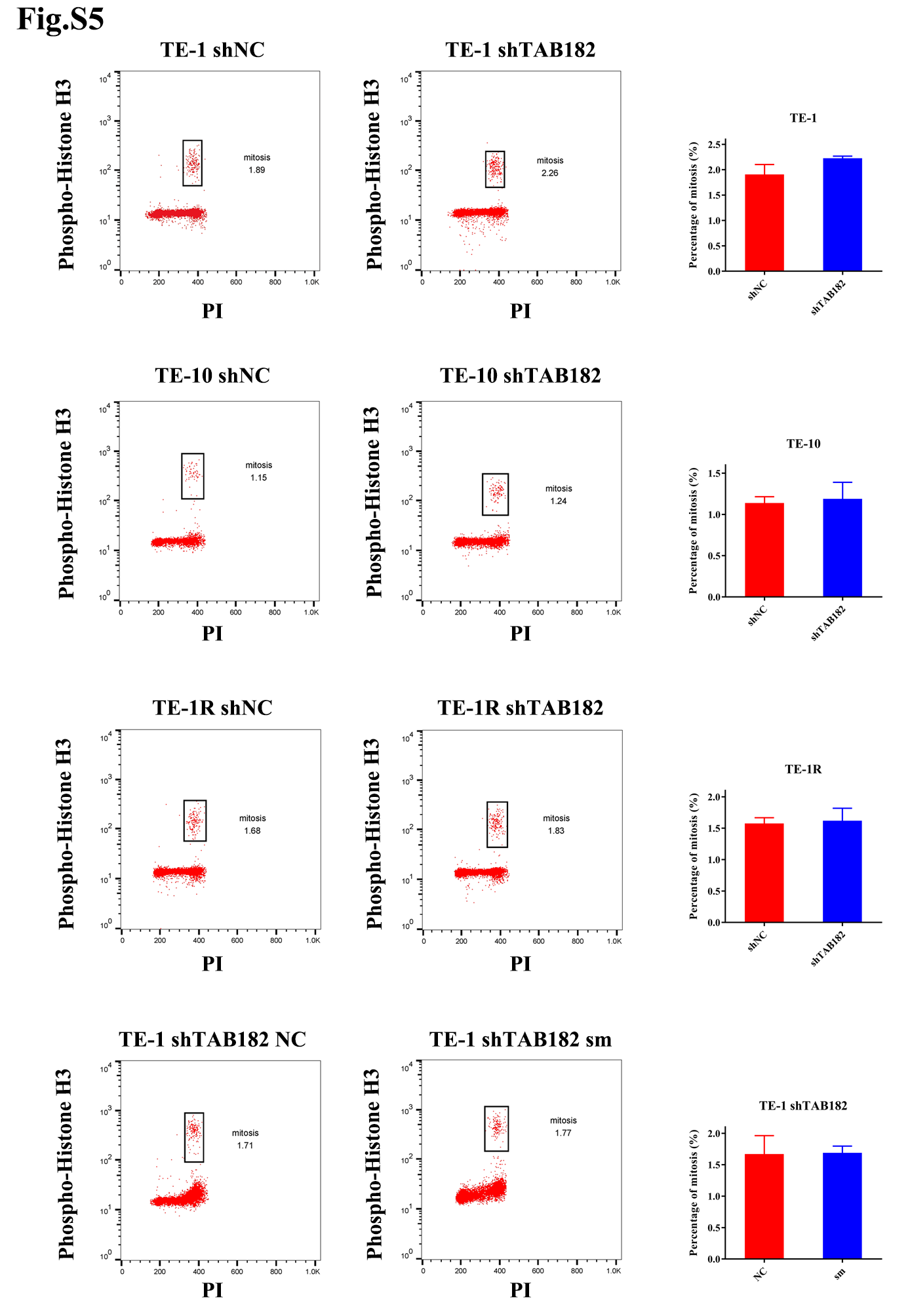

Supplement: Supplementary file 5 — Fig S5 [file CAM4-10-3101-s004.tif]

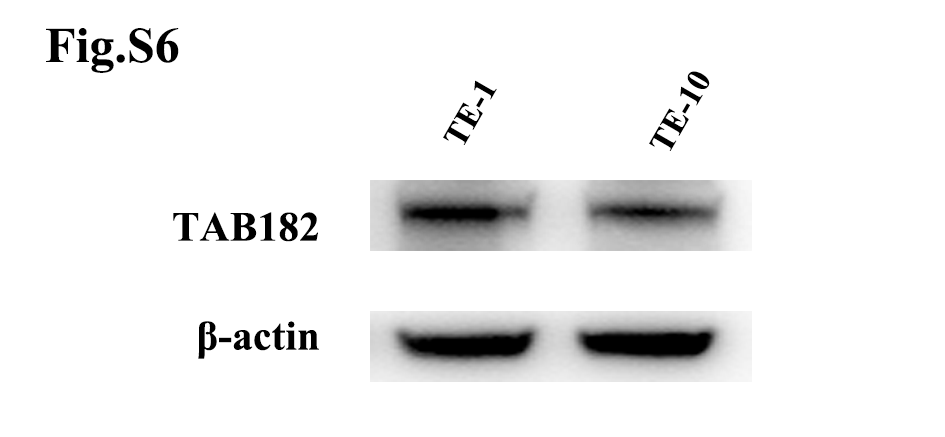

Supplement: Supplementary file 6 — Fig S6 [file CAM4-10-3101-s003.tif]

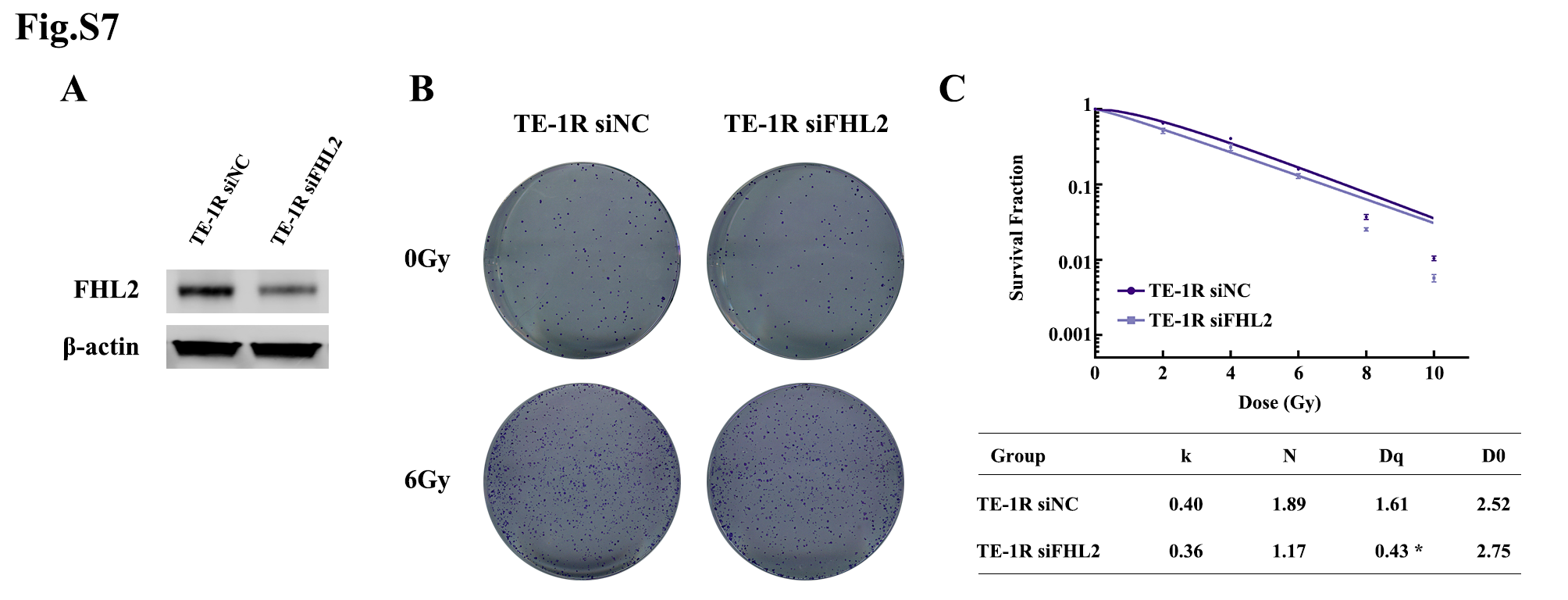

Supplement: Supplementary file 7 — Fig S7 [file CAM4-10-3101-s006.tif]
