# Supplementary material for: Elevated TAB182 enhances the radioresistance of esophageal squamous cell carcinoma through G2‐M checkpoint modulation
Source: Cancer Med. 2021 Mar 30;10(9):3101–12. doi: 10.1002/cam4.3879 (PMC8085956; doi:10.1002/cam4.3879)
Supplement: Supplementary file 8 — Supplementary Material [file CAM4-10-3101-s005.docx]

**Supplemental materials**

**Sequence of shTAB182.**

Sense: GCCAAGACCAGAGUAAAGUTT

Antisense: ACUUUACUCUGGUCUUGGCTT

Sequence of shTAB182 sm:

| 1 |  | ATGGATTACA AGGATGACGA CGATAAGAAA GTGTCTACTC TCAGGGAAAG CTCAGCCATG |  | 60 |
| --- | --- | --- | --- | --- |
| 61 |  | GCTTCCCCAC TGCCCCGGGA GATGGAGGAG GAGCTGGTGC CTACTGGCTC TGAGCCAGGT |  | 120 |
| 121 |  | GACACTCGGG CCAAACCCCC TGTCAAGCCC AAACCCCGGG CCCTGCCTGC CAAGCCAGCC |  | 180 |
| 181 |  | CTGCCTGCCA AACCCAGCCT GCTGGTGCCT GTTGGGCCTC GGCCTCCCCG GGGTCCCCTG |  | 240 |
| 241 |  | GCTGAGTTGC CTTCTGCCAG GAAGATGAAC ATGCTGGCAG GACCCCAGCC CTATGGTGGC |  | 300 |
| 301 |  | AGCAAGCGCC CCCTTCCCTT TGCACCAAGG CCTGCGGTTG AGGCCTCCAC TGGAGGAGAA |  | 360 |
| 361 |  | GCCACCCAAG AGACTGGGAA AGAGGAGGCT GGGAAAGAGG AGCCACCCCC TTTGACACCC |  | 420 |
| 421 |  | CCAGCTCGAT GTGCAGCCCC AGGGGGTGTA CGGAAGGCCC CTGCCCCTTT CCGCCCAGCC |  | 480 |
| 481 |  | TCAGAGCGCT TCGCGGCCAC CACGGTGGAA GAGATCCTGG CCAAGATGGA GCAGCCTCGG |  | 540 |
| 541 |  | AAGGAGGTCC TTGCCAGCCC CGACCGCCTG TGGGGTTCCC GCCTCACCTT TAACCACGAT |  | 600 |
| 601 |  | GGCAGCTCGC GATATGGCCC CAGGACCTAT GGCACGACCA CTGCTCCCAG GGATGAGGAT |  | 660 |
| 661 |  | GGCAGCACCC TCTTCAGGGG ATGGTCCCAG GAGGGGCCAG TAAAGTCTCC AGCAGAGTGC |  | 720 |
| 721 |  | CGGGAAGAGC ACAGCAAGAC CCCTGAGGAG AGGAGCCTTC CTTCCGACCT GGCCTTCAAC |  | 780 |
| 781 |  | GGGGACCTGG CTAAGGCAGC CAGCTCGGAG CTACCTGCTG ATATTTCCAA GCCCTGGATT |  | 840 |
| 841 |  | CCCTCAAGTC CAGCCCCCTC CTCAGAGAAT GGAGGCCCTG CCAGCCCAGG CCTCCCCGCA |  | 900 |
| 901 |  | GAAGCCTCAG GCTCAGGCCC TGGCTCTCCC CATCTTCACC CGCCTGATAA GAGTTCTCCC |  | 960 |
| 961 |  | TGCCACTCAC AGCTTCTGGA AGCCCAGACT CCTGAAGCTT CCCAGGCTTC TCCCTGCCCC |  | 1020 |
| 1021 |  | GCTGTGACTC CATCAGCTCC AAGTGCAGCC CTGCCTGACG AGGGCTCCCG CCACACCCCC |  | 1080 |
| 1081 |  | AGCCCGGGGC TCCCTGCCGA GGGGGCTCCA GAGGCCCCCA GACCCAGCAG CCCACCCCCT |  | 1140 |
| 1141 |  | GAGGTCTTGG AGCCCCATAG CCTGGATCAG CCCCCTGCCA CCTCACCCCG GCCCCTGATC |  | 1200 |
| 1201 |  | GAGGTGGGTG AGTTGCTGGA TCTCACTCGG ACGTTTCCAT CTGGCGGGGA GGAGGAGGCC |  | 1260 |
| 1261 |  | AAGGGTGACG CACACCTCCG CCCCACCAGC CTGGTTCAGC GCCGATTCTC TGAAGGTGTG |  | 1320 |
| 1321 |  | CTCCAGTCAC CCAGTCAGGA CCAGGAGAAG CTGGGGGGCT CGCTGGCTGC CCTGCCCCAA |  | 1380 |
| 1381 |  | GGCCAGGGGA GCCAGTTGGC CCTGGATCGT CCCTTTGGGG CAGAGTCCAA CTGGAGCTTA |  | 1440 |
| 1441 |  | TCACAGTCCT TCGAATGGAC CTTCCCCACG AGGCCCTCGG GTCTGGGCGT GTGGCGGCTG |  | 1500 |
| 1501 |  | GACTCCCCGC CTCCCTCCCC CATCACTGAA GCCAGTGAGG CCGCCGAGGC TGCTGAGGCT |  | 1560 |
| 1561 |  | GGCAACTTGG CCGTTTCCAG CAGGGAAGAA GGAGTGTCTC AGCAGGGGCA AGGGGCTGGG |  | 1620 |
| 1621 |  | TCAGCTCCAA GTGGGTCAGG AAGTTCCTGG GTGCAGGGGG ATGATCCAAG CATGTCCCTC |  | 1680 |
| 1681 |  | ACCCAGAAGG GCGATGGGGA GAGTCAACCT CAATTCCCAG CTGTTCCCCT TGAGCCCCTG |  | 1740 |
| 1741 |  | CCTACAACTG AGGGCACACC TGGATTACCT TTGCAGCAGG CAGAGGAGAG ATACGAGTCG |  | 1800 |
| 1801 |  | CAGGAGCCCT TGGCTGGACA GGAGTCCCCT CTCCCCCTGG CTACCAGGGA GGCAGCCTTG |  | 1860 |
| 1861 |  | CCCATCCTGG AGCCAGTCCT GGGGCAGGAG CAGCCAGCAG CCCCTGACCA GCCCTGTGTT |  | 1920 |
| 1921 |  | CTCTTTGCTG ATGCCCCTGA GCCTGGACAG GCACTGCCTG TTGAGGAGGA GGCCGTGACC |  | 1980 |
| 1981 |  | CTAGCCCGGG CTGAGACCAC CCAAGCCAGG ACAGAGGCTC AAGACTTGTG TAGGGCATCC |  | 2040 |
| 2041 |  | CCCGAGCCTC CAGGCCCTGA AAGCAGCTCC CGCTGGCTGG ACGACCTCCT GGCTTCACCA |  | 2100 |
| 2101 |  | CCACCCAGTG GTGGCGGTGC AAGGCGGGGA GCTGGAGCTG AGCTGAAGGA CACACAGTCC |  | 2160 |
| 2161 |  | CCAAGTACCT GCTCTGAGGG ACTCCTTGGC TGGTCCCAGA AAGATCTGCA GAGTGAATTT |  | 2220 |
| 2221 |  | GGGATCACAG GAGACCCACA GCCCAGCAGT TTCAGTCCTT CCAGCTGGTG TCAAGGTGCT |  | 2280 |
| 2281 |  | TCTCAGGACT ATGGCCTTGG GGGTGCAAGC CCTAGAGGAG ACCCAGGTCT CGGAGAGAGG |  | 2340 |
| 2341 |  | GACTGGACCA GCAAGTATGG GCAAGGAGCA GGGGAAGGGA GCACCAGGGA GTGGGCCAGC |  | 2400 |
| 2401 |  | AGGTGTGGCA TCGGCCAGGA GGAGATGGAG GCCAGCAGCA GCCAGGATCA AAGTAAAGTG |  | 2460 |
| 2461 |  | TCTGCCCCAG GGGTGCTCAC AGCCCAGGAC CGGGTAGTTG GAAAGCCAGC CCAGCTTGGC |  | 2520 |
| 2521 |  | ACTCAGCGGA GCCAGGAGGC AGATGTTCAG GACTGGGAGT TCAGAAAGAG GGATTCCCAG |  | 2580 |
| 2581 |  | GGCACTTACT CCAGCCGGGA TGCAGAACTC CAGGACCAGG AATTCGGAAA GAGAGATTCA |  | 2640 |
| 2641 |  | CTGGGTACCT ACAGTAGTCG AGATGTAAGC CTTGGGGACT GGGAATTTGG GAAGAGAGAT |  | 2700 |
| 2701 |  | TCTCTGGGTG CTTATGCCAG CCAAGATGCC AACGAGCAGG GCCAAGATTT GGGGAAGAGG |  | 2760 |
| 2761 |  | GACCACCATG GTAGGTACAG CAGCCAGGAT GCCGATGAGC AGGACTGGGA GTTTCAGAAG |  | 2820 |
| 2821 |  | AGAGATGTGT CACTCGGCAC CTATGGCAGC CGGGCTGCGG AGCCACAGGA ACAGGAGTTT |  | 2880 |
| 2881 |  | GGGAAGAGCG CTTGGATAAG GGACTACAGC AGTGGTGGCA GCTCCAGGAC CCTTGACGCC |  | 2940 |
| 2941 |  | CAGGACAGAA GCTTTGGAAC GAGACCCCTG AGCTCTGGGT TCAGCCCCGA GGAAGCCCAG |  | 3000 |
| 3001 |  | CAACAGGATG AGGAATTTGA GAAGAAGATT CCAAGTGTGG AAGACAGCCT TGGAGAGGGC |  | 3060 |
| 3061 |  | AGCAGGGATG CTGGCCGGCC AGGAGAGAGA GGATCCGGGG GCTTGTTCAG TCCTAGCACT |  | 3120 |
| 3121 |  | GCCCACGTGC CGGATGGGGC ACTCGGGCAG AGAGACCAGA GCAGCTGGCA AAACAGTGAT |  | 3180 |
| 3181 |  | GCTAGCCAGG AGGTGGGAGG GCATCAGGAG AGACAGCAGG CAGGGGCTCA GGGCCCTGGC |  | 3240 |
| 3241 |  | AGTGCTGACC TGGAAGATGG GGAGATGGGA AAGCGAGGCT GGGTCGGTGA GTTTAGCCTC |  | 3300 |
| 3301 |  | AGTGTTGGCC CCCAGCGAGA GGCAGCATTT AGCCCAGGGC AGCAGGACTG GAGCCGGGAC |  | 3360 |
| 3361 |  | TTCTGCATCG AGGCCAGTGA GAGGAGCTAT CAGTTTGGCA TCATTGGCAA CGACAGAGTG |  | 3420 |
| 3421 |  | AGTGGTGCTG GCTTTAGCCC TTCTAGCAAG ATGGAAGGTG GTCACTTTGT GCCTCCTGGG |  | 3480 |
| 3481 |  | AAGACCACAG CTGGCTCGGT GGACTGGACT GACCAGCTGG GTCTCAGGAA CTTGGAAGTG |  | 3540 |
| 3541 |  | TCCAGCTGTG TGGGTTCTGG GGGCTCGAGC GAGGCCAGGG AGAGTGCCGT GGGACAGATG |  | 3600 |
| 3601 |  | GGCTGGTCAG GTGGCCTGAG CTTGAGAGAC ATGAACCTGA CCGGCTGTTT GGAAAGTGGA |  | 3660 |
| 3661 |  | GGGTCTGAAG AGCCGGGGGG AATCGGAGTT GGGGAGAAGG ACTGGACTTC TGATGTTAAT |  | 3720 |
| 3721 |  | GTGAAGAGCA AAGATTTGGC TGAGGTCGGG GAGGGAGGAG GCCACAGCCA GGCCAGAGAG |  | 3780 |
| 3781 |  | AGTGGCGTGG GGCAGACTGA CTGGTCAGGT GTGGAGGCCG GAGAGTTCCT TAAATCAAGG |  | 3840 |
| 3841 |  | GAGCGTGGAG TTGGACAGGC AGACTGGACA CCTGACCTTG GGCTGAGAAA CATGGCCCCA |  | 3900 |
| 3901 |  | GGGGCAGTCT GCAGTCCTGG AGAGTCCAAA GAGCTTGGGG TGGGCCAGAT GGACTGGGGT |  | 3960 |
| 3961 |  | AACAATCTGG GCCTGAGGGA TTTGGAGGTG ACCTGTGACC CAGACTCTGG AGGTTCTCAG |  | 4020 |
| 4021 |  | GGGCTACGGG GATGTGGAGT GGGGCAGATG GACTGGACCC AGGACTTGGC GCCCCAGAAT |  | 4080 |
| 4081 |  | GTGGAGCTCT TTGGGGCTCC AAGTGAAGCC AGGGAGCATG GGGTGGGCGG GGTGAGCCAG |  | 4140 |
| 4141 |  | TGCCCAGAGC CCGGCCTGAG GCACAATGGC AGCTTGTCTC CTGGCCTGGA GGCCAGAGAC |  | 4200 |
| 4201 |  | CCCTTGGAGG CCAGGGAGCT GGGGGTTGGT GAGACAAGTG GGCCAGAGAC CCAGGGTGAA |  | 4260 |
| 4261 |  | GATTACTCCT CGTCTTCCTT GGAGCCACAC CCTGCAGACC CTGGAATGGA GACAGGAGAA |  | 4320 |
| 4321 |  | GCCCTCAGCT TCGGAGCAAG CCCTGGCAGG TGCCCGGCCC GCCCCCCACC CTCCGGCTCC |  | 4380 |
| 4381 |  | CAGGGCCTGC TGGAGGAGAT GCTGGCAGCC AGCAGCTCCA AGGCGGTGGC TCGGAGGGAG |  | 4440 |
| 4441 |  | TCAGCGGCCT CGGGCCTTGG GGGCCTGTTG GAGGAGGAAG GAGCCGGGGC AGGTGCTGCC |  | 4500 |
| 4501 |  | CAAGAGGAGG TGCTGGAGCC TGGCAGGGAC TCTCCACCCT CCTGGAGGCC GCAGCCTGAT |  | 4560 |
| 4561 |  | GGTGAGGCCA GCCAGACAGA AGACGTGGAT GGCACCTGGG GCTCTTCAGC AGCCAGGTGG |  | 4620 |
| 4621 |  | AGCGATCAGG GGCCAGCACA GACTTCTCGG CGACCCTCCC AAGGCCCTCC TGCCAGATCC |  | 4680 |
| 4681 |  | CCCAGTCAGG ACTTCTCCTT CATTGAGGAC ACCGAGATCC TCGACAGTGC CATGTATCGG |  | 4740 |
| 4741 |  | AGCCGTGCCA ACTTGGGGCG CAAGCGTGGG CACCGGGCCC CGGTCATTCG GCCTGGGGGT |  | 4800 |
| 4801 |  | ACCTTGGGCC TGTCGGAGGC AGCAGACTCG GATGCACACC TGTTCCAGGA CTCTACAGAG |  | 4860 |
| 4861 |  | CCACGGGCAT CTCGGGTGCC ATCTTCAGAT GAAGAGGTAG TGGAGGAACC TCAGAGCCGC |  | 4920 |
| 4921 |  | CGGACACGGA TGTCGTTGGG CACCAAGGGG CTGAAAGTCA ACCTCTTTCC TGGCCTGAGC |  | 4980 |
| 4981 |  | CCCTCAGCCC TGAAGGCCAA GCTGCGCCCC CGGAATCGCT CAGCTGAGGA GGGAGAGCTG |  | 5040 |
| 5041 |  | GCTGAGAGCA AGTCGAGCCA GAAGGAGTCC GCGGTCCAGC GTTCGAAATC CTGCAAGGTC |  | 5100 |
| 5101 |  | CCAGGACTGG GAAAGCCCCT CACGTTACCT CCCAAGCCAG AGAAATCCTC AGGGTCAGAA |  | 5160 |
| 5161 |  | GGATCGTCGC CCAACTGGCT TCAAGCCCTG AAACTGAAGA AGAAGAAGGT CTGA |  | 5214 |
